# Benefits for Plants in Ant-Plant Protective Mutualisms: A Meta-Analysis

**DOI:** 10.1371/journal.pone.0014308

**Published:** 2010-12-22

**Authors:** Matthew D. Trager, Smriti Bhotika, Jeffrey A. Hostetler, Gilda V. Andrade, Mariano A. Rodriguez-Cabal, C. Seabird McKeon, Craig W. Osenberg, Benjamin M. Bolker

**Affiliations:** 1 Florida Museum of Natural History, University of Florida, Gainesville, Florida, United States of America; 2 School of Natural Resources and Environment, University of Florida, Gainesville, Florida, United States of America; 3 Department of Biology, University of Florida, Gainesville, Florida, United States of America; 4 Department of Wildlife Ecology and Conservation, University of Florida, Gainesville, Florida, United States of America; 5 Departamento de Biologia, Universidade Federal do Maranhão, São Luís, Maranhão, Brazil; 6 Department of Ecology and Evolutionary Biology, University of Tennessee, Knoxville, Tennessee, United States of America; Royal Holloway University of London, United Kingdom

## Abstract

Costs and benefits for partners in mutualistic interactions can vary greatly, but surprisingly little is known about the factors that drive this variation across systems. We conducted a meta-analysis of ant-plant protective mutualisms to quantify the effects of ant defenders on plant reproductive output, to evaluate if reproductive effects were predicted from reductions in herbivory and to identify characteristics of the plants, ants and environment that explained variation in ant protection. We also compared our approach with two other recent meta-analyses on ant-plant mutualisms, emphasizing differences in our methodology (using a weighted linear mixed effects model) and our focus on plant reproduction rather than herbivore damage. Based on 59 ant and plant species pairs, ant presence increased plant reproductive output by 49% and reduced herbivory by 62%. The effects on herbivory and reproduction within systems were positively correlated, but the slope of this relationship (0.75) indicated that tolerance to foliar herbivory may be a common plant response to absence of ant guards. Furthermore, the relationship between foliar damage and reproduction varied substantially among systems, suggesting that herbivore damage is not a reliable surrogate for fitness consequences of ant protection. Studies that experimentally excluded ants reported a smaller effect of ant protection on plant reproduction than studies that relied upon natural variation in ant presence, suggesting that study methods can affect results in these systems. Of the ecological variables included in our analysis, only plant life history (i.e., annual or perennial) explained variation in the protective benefit of mutualistic ants: presence of ants benefitted reproduction of perennials significantly more than that of annuals. These results contrast with other quantitative reviews of these relationships that did not include plant life history as an explanatory factor and raise several questions to guide future research on ant-plant protection mutualisms.

## Introduction

Ant-plant protection mutualisms are model systems for examining the evolution and maintenance of mutualistic relationships [1]–[3], plant defense strategies [4]–[6], species coexistence [7]–[10] and multitrophic interactions [11], [12]. These relationships most commonly involve an exchange of resources and services in which plants produce food rewards or housing for ants that then defend the host plant against herbivores, pathogens and encroaching vegetation. Symbiotic myrmecophytes – plant species that endogenously produce small chambers called domatia in which ant colonies reside – have more specific associations with ants and are thought to be more dependent on ant protection than plants that provide only food rewards for ant guards (i.e., nonsymbiotic myrmecophiles) [2], [13], [14]. In some symbiotic ant-plant relationships, the ant and plant species have coevolved to the point where the interaction is considered obligate for one or both partners [2], [15]. However, within individual ant-plant systems, the costs and benefits for both partners can be affected by many factors. For example, the nature and strength of the interactions may depend upon the ontogeny of the host plant or ant colony [16], [17], nutrient or light availability [18]–[20], herbivore pressure [21], [22] or relationships with other organisms, such as ant-tended herbivores [23], [24] or pollinators [3], [25], [26]. Despite numerous studies testing various ant, plant and environmental characteristics that affect individual ant-plant protection mutualisms, we still know relatively little about the traits that drive patterns in the efficacy of ant defense across systems.

Two recent meta-analyses quantified responses of ant-plants to the absence of ants across multiple systems and explored the effects of different traits on the magnitude of ant protection for their host plants [27], [28]. Both meta-analyses included studies of effects on herbivory and plant reproduction; however, studies on plant reproduction were relatively scarce and only a few of the summarized studies reported effects on both herbivory and reproduction. In their study, Chamberlain and Holland [27] found that ant presence had a larger effect on herbivory than on plant “performance” (consisting primarily of studies of reproduction), but effects on herbivory versus reproduction were not correlated among studies that measured both responses. This finding suggests that the most commonly measured benefit for plants of ant guards, defense against herbivory, is a poor predictor of effects on plant reproduction (*contra* [2]). Additionally, both Chamberlain and Holland [27] and Rosumek et al. [28] found several ecological variables that affected the magnitude of ant protection against herbivory, but fewer factors that affected plant reproductive output. If these results are generalizable across systems, it should inform how future experiments on ant-plant mutualistic system are conducted and interpreted and might provide insight into plant strategies for resource allocation among anti-herbivore defense, growth and reproduction.

Because methodological variation can dramatically affect results and is common among quantitative syntheses addressing similar questions (e.g., [29], [30]), it is important to evaluate the robustness of meta-analytic conclusions that employ different criteria for selection of studies and different statistical approaches [31], [32]. For example, assessing and accounting for non-independence among data is an important and challenging aspect of conducting meta-analyses [31]. A single ecological study might report results from work conducted over several seasons or in multiple locations on a single species, or different research groups may separately report results on a single system [29], [33]. Oversampling a particular species in a meta-analytic data set (i.e., via non-independence among samples) can introduce biases into the analyses and lead to incorrect inferences about the role of predictor variables such as organismal or environmental traits [34]. Conversely, pooling all data from multiple studies on a single taxon prior to analysis may be overly conservative and may preclude examination of potentially important factors that differ among studies conducted on the same species. Hierarchical approaches can satisfactorily account for heterogeneity within or among systems, but such techniques are not commonly applied to ecological meta-analyses [31].

We conducted a meta-analysis to quantify the effects of ant protection on herbivore damage and plant reproductive output and then tested the effects of plant, ant and environmental characteristics on the magnitude of the reproductive benefit conferred by ants on their host plants. We addressed several specific hypotheses that have been proposed in the literature and evaluated the effects of other explanatory variables that we thought could contribute to variation in the protective effect among systems (Table 1).

**Table 1:** Plant, ant, environmental, and study characteristics included in the meta-analysis, and hypotheses related to the potential effects of those variables.

Variable	Type (values)	Hypotheses
**Plant characteristics**		
Life history	Categorical (annual or perennial)	Annual plants will benefit less from ant defense than perennial plants because the annuals should invest less in defense [55].
Domatia	Categorical (present or absent)	Plants that produce domatia will benefit more from ant presence [2].
Location of extrafloral nectaries (EFN)	Categorical (vegetative or reproductive)	The functions of EFN on reproductive structures differ among systems: 1. ants visiting such EFN could deter pollinators, thereby reducing reproductive output [25], [47], 2. EFN on reproductive parts could attract ants or non-ant predators or parasitoids that deter or predate herbivores or seed predators, thereby increasing plant reproductive output [56], [57]. Given conflicting evidence regarding the function of EFN on vegetative versus reproductive structures, we had no strong reason to expect a particular effect of EFN location on ant effects on plant reproduction.
Honeydew-producing trophobionts	Categorical (present or absent)	May be costly to the plant [23], [58], thereby reducing reproductive output when ants are removed.
Growth form	Categorical (herb, shrub, tree, vine, epiphyte, cactus)	No a priori hypothesis regarding direct effects, although growth form may be correlated with plant reward structure or life history to influence ant protection.
**Ant characteristics**		
Species diversity	Continuous (1/n)	Protective benefits of ants will decrease with the number of ant species associated with plant species [62]; may be confounded with presence of domatia since domatia-bearing plants have closer relationships with fewer ant partners [2]. (Note: We used the inverse of ant species richness because it is a better measure for diffusion of mutualism specificity).
Subfamily of most abundant ant	Categorical	No a priori hypothesis, but phylogenetic covariates could affect mutualism function
Size of most abundant ant	Continuous (body length in mm)	Large ants will confer greater protective benefits, at least for myrmecophilic plants that do not produce domatia (reviewed in [14], but see [68]); relationship may be dependent on presence of domatia if ants associated with domatia-bearing plants are smaller.
**Environmental characteristics**		
Habitat type	Categorical (forest, open, desert/beach)	No a priori hypothesis; included primarily to test in interaction with precipitation to more specifically indicate abiotic factors.
Mean annual precipitation	Continuous (mm)	Herbivore pressure is stronger in tropical forests [66], so the protective effect of ants should increase with precipitation, in forested habitats or as a function of an interaction between precipitation and habitat type.
**Study characteristics**		
Study type	Categorical (experimental or observational)	No a priori hypothesis, but differences in design could influence derived effect sizes.

Our questions and methodological approach differed from previous meta-analyses on ant-plant protection systems in four key ways: 1) we included only studies examining ant effects on plant reproductive output, a better proxy measure of fitness consequences than ant effects on herbivory or herbivore abundance [35]; 2) we included more characteristics of the ant and plant species, including plant life history (a trait not examined in either previous meta-analysis despite its importance for plant defense strategies); 3) we explicitly examined correlations among predictor variables to aid in our interpretations and avoid spurious results; and 4) we applied a flexible and powerful hierarchical statistical approach to better deal with non-independence of multiple results from the same plant species and to allow simultaneous analysis of more than one predictor variable. Consequently, our results generate new insights into the sources of variation in the benefits that plants gain from ant presence and provide direction for future studies on ant-plant protection mutualisms.

## Methods

### Literature search and data extraction

We conducted an extensive literature search to find peer-reviewed studies of ant-plant protection mutualisms (Appendix S1). From the approximately 400 papers produced by our searches, only 31 quantified plant reproductive output in the presence and absence of presumably protective ants and provided sufficient information for calculating effect sizes. Of these, 24 studies experimentally removed or excluded ants from experimental plants and 7 compared plants that naturally varied in the presence or absence of ants. From these papers, we extracted means and variances of reproductive output and foliar damage of host plants (Appendix S1), as well as information about the ants, plants and environment (Table 1). Because not all papers described the plant species in their study systems as obligate or facultative, we tested the effects of several factors that are generally considered to be indicative of the degree of dependency between plants and their ant guards (e.g., domatia production, number of ant partners, perennial or annual life history). We excluded studies in which the ant species had been previously identified as a parasite on the host plant (e.g., [36]–[38]). For studies with time series data, we used only the data from the final time period, and for studies that included data for more than one reproductive stage (e.g., flowers, fruits and seeds), we extracted data only from the most advanced stage (on the assumption that it was a better proxy for plant fitness). Several papers contained information for more than one ant species. In those cases we calculated an effect size for each ant and plant pair.

### Effect sizes

We used a log response ratio to quantify the effect of ants on plant reproduction [29], [30], [39]:
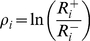
(1)where *ρ_i_* is the reproductive effect size and *R_i_^+^* and *R_i_^−^* represent reproductive output with and without ants, respectively, for the *i* ^th^ study. Variance of the effect was approximated with the delta method [40], [41]:
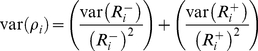
(2)We defined a similar effect size for foliar herbivore damage (designated here as *η*), but we modified Equation 1 by inverting the ant presence and absence treatments so that the sign of both reproductive and herbivory effect sizes would be positive if ant presence benefited plants. As such, we calculated the herbivory effect size as
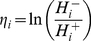
(3)with variance of the effect size estimated as:
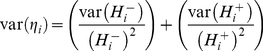
(4)where *η_i_* is the herbivory effect size and *H_i_^+^* and *H_i_^−^* represent the amount of foliar damage with and without ants, respectively, for the *i* ^th^ study.

For multiple results from the same ant and plant species pairs that did not differ in any of the other covariates listed in Table 1, we calculated a variance-weighted average effect size to avoid inflating their influence in the analysis:
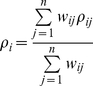
(5)with variance:
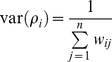
(6)where *ρ_ij_* is the effect size for the *j* ^th^ site or year for the *i* ^th^ study, *w_ij_* is the weight for the *j* ^th^ site or year (*w_ij_* = 1/var(*ρ_ij_*)) and *ρ_i_* is the average effect size.

### Data analysis

We tested the relationship between plant reproduction and foliar damage with a weighted correlation analysis using studies that provided data on both responses. We minimized a weighted sum of squares, corresponding to a negative log-likelihood for a linear model with known, normally-distributed errors in both *x* and *y*:
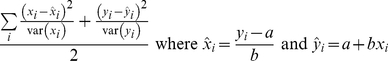
(7)We used the slope and intercept terms from an ordinary least-squares linear regression of *y* on *x* as the starting values for the minimization function, and calculated 95% confidence intervals for the slope and intercept terms from the likelihood profile. We performed this analysis using the R Language and Environment for Statistical Computing [42] with the package bbmle (v. 0.9.0) for maximum likelihood estimation [41], [43] (Appendix S2).

Before testing the effects of ecological traits on the effect of ant presence on plant reproduction, we tested for associations among the predictor variables. We used Fisher's exact test to examine relationships among the plant-level categorical variables (i.e., life history, habitat type, location of extrafloral nectaries, the presence of domatia or honeydew-producing trophobionts, and whether the data were derived from experimental or observational studies), and Pearson product-moment correlation to test for relationships between the continuous, individual-study-level variables (i.e., precipitation and number of ant species) and between these variables and the binary plant-level categorical variables. We did not test for relationships between continuous variables and categorical variables with three or more levels. Elucidating patterns among mutualism traits allowed us to better interpret statistical models that included potentially correlated predictor variables.

To test hypotheses regarding the effects of plant, ant and environmental characteristics on the degree to which ant protection increased plant reproduction, we used a weighted linear mixed effects model fitted by restricted maximum likelihood [44]. In these analyses, the effect sizes of each ant and plant pair was weighted by the inverse of its total variance, a procedure that calculated the influence of individual primary studies on our results based on how precisely they estimated the response variables [39]. The main source of non-independence among samples in our data set arises from multiple effect sizes for the same plant species, albeit with different ant species. Thus, we incorporated the possible plant species effect by including plant species as a random grouping variable in all of these analyses. All other ant, plant and environmental predictor variables listed in Table 1 were defined in the model as fixed effects. Due to correlations among many variables or missing combinations of factors, we tested for significant interactions among variables only when the interaction terms were applicable to specific hypotheses we intended to evaluate *a priori*. We conducted all mixed effects models analyses with the package nlme (v. 3.1–93) [45] in the R Language and Environment for Statistical Computing [42] following model specification and interpretation protocols described by Pinheiro and Bates [44] (Appendix S2).

## Results

**Figure 1:**
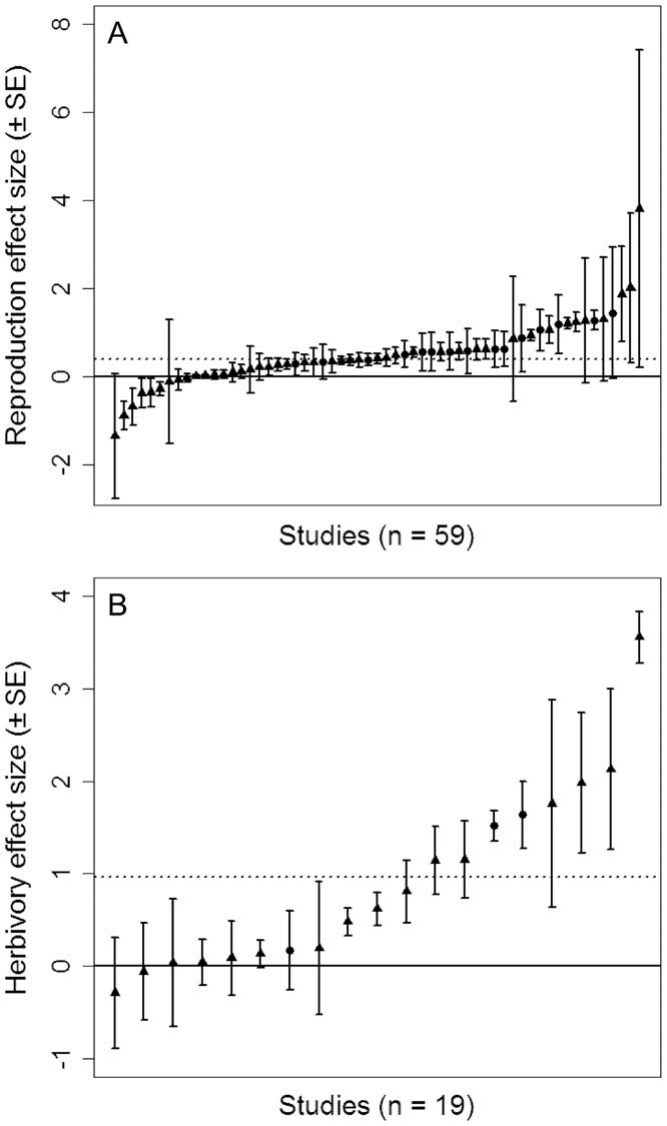
Effect sizes (means ±95% confidence intervals) for responses of (A) plant reproduction and (B) herbivore damage to ant presence, ordered by magnitude. For both panels, the solid line indicates no effect (log-ratio  = 0) and the dashed line indicates the weighted mean effect size. Circles represent observational studies and triangles represent experimental studies; note that the *y*-axis scales are different for (A) and (B).

Our analyses included reproductive data from 31 journal articles, comprising 28 plant species and 59 ant and plant species combinations (Table S1). Of these studies, 19 also included data on foliar herbivory. The effect sizes varied substantially but, on average, ant presence increased plant reproductive output by 49% (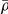 = 0.40±0.074 SE) and decreased foliar herbivory by 62% (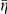 = 0.96±0.23 SE: Fig. 1).

There were relatively few significant correlations among the ant, plant and environmental predictor variables, thus reducing concern about spurious interpretation of results. Among the 28 plant species in our study, those that produced domatia had significantly fewer associated ant species (t_26_ = −2.09, P = 0.046). The presence of honeydew-producing trophobionts tended by the ants (e.g., coccoids or aphids) was positively correlated with both annual precipitation (t_57_ = 2.39, P = 0.020) and the location of extrafloral nectaries on the plant reproductive structures (t_57_ = 2.62, P = 0.011). Experimental studies were more likely to be conducted in systems with high annual precipitation (t_57_ = 3.20, P = 0.0023) and on those plants that produced EFN on reproductive structures (Fisher's Exact Test, P = 0.0009). No other variables were significantly correlated, although this may have been due to low power arising from small sample sizes. Additionally, there were no annual plants with domatia, making it impossible to meaningfully assess correlations and difficult to interpret the individual results of these two factors (see below).

Of the 59 reproduction effect sizes, 44 were from experimental studies and 15 were from observational studies. Both the experimental and observational groups had significantly positive mean effect sizes (Experimental: 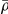
_Exp_ = 0.32±0.079 SE, t_26_ = 3.89, P = 0.0003; Observational: 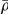
_Obs_ = 0.66±0.15 SE, t_26_ = 4.39, P<0.0002), indicating beneficial effects of ant presence for plant reproduction regardless of study type. Although potentially important, the two-fold difference in effect size between experimental and observational studies was only marginally statistically significant (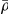
_Obs_ - 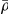
_Exp_ = 0.34±0.17, t_26_ = −1.98, P = 0.058). However, because study type accounted for large amounts of variation in effect sizes, and because the inclusion of other variables often led to a significant effect of study type (see below), we included it as a factor in all analyses to increase the power of detecting effects of the ecological variables. We initially included interactions between the focal variable and study type in the models, but the interaction term was never significant, so we removed it from the final models. We summarize ecological effects using marginal means averaged over the two levels of study type.

### Effects of ant protection on herbivory and plant reproduction

**Figure 2:**
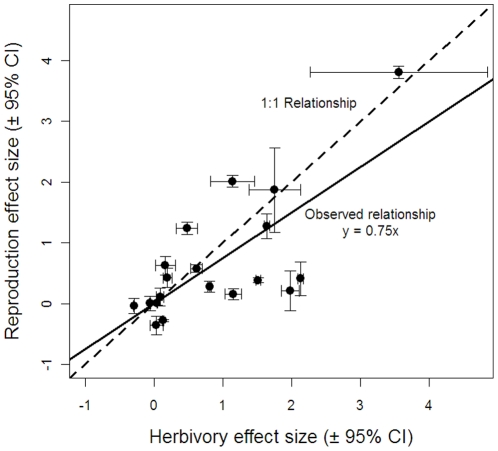
Relationship between the effect sizes for herbivory and reproductive output. Means (±95% CI) for both effect sizes are presented for the 19 studies that contained data on both herbivore damage and reproductive output. The dashed line indicates a hypothetical 1∶1 relationship; the solid line indicates the observed relationship (see Equation 5 for weighted correlation procedure). The slope of the relationship was significantly less than 1 (Maximum Likelihood Estimate  = 0.75, 95% CI  = 0.71 to 0.79).

Effect sizes for foliar damage and reproductive output were positively correlated for the 19 studies that included both responses to ant protection, but the slope of the correlation was significantly less than 1 (Fig. 2). This slope suggests that ant absence had a smaller mean effect on plant reproductive output than would be expected based on the corresponding foliar herbivory effect size. However, the relationship was inconsistent among studies, with some falling appreciably above and others below the expected 1∶1 line. Indeed, despite the overall significant positive correlation, the 95% confidence intervals for herbivory and reproductive effects did not overlap each other for 13 of 19 studies, further demonstrating that measuring herbivory alone can lead to misleading conclusions on the effects of ant guards on short-term estimates of plant fitness.

### Effects of plant characteristics on mutualism strength

Four plant species in our study, comprising 9 of the 59 ant and plant combinations, had annual life cycles. Ant presence had a significantly larger reproduction effect size for perennial plants than for annual plants (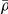
_perennial_ - 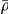
_annual_ = 0.38±0.13 SE; F_1, 25_ = 9.72, P = 0.0045), with experimental studies producing smaller effect sizes than observational studies in the two-factor analysis regardless of plant life history (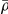
_obs_ - 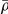
_exp_ = 0.33±0.15 SE; F_1, 25_ = 4.59, P = 0.042). Averaging across experimental and observational studies, our model predicts an 18% increase in reproductive output for annual plants when ants are present (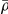
_annual_ = 0.17±0.17 SE) and a 73% increase in reproductive output for perennial plants when ants are present (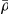
_perennial_ = 0.55±0.13 SE).

Six plant species in our study, comprising 10 ant and plant species combinations, produced domatia occupied by ant guards. The effects of ant presence for plant reproduction did not differ significantly according to production of domatia (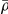
_domatia_ - 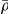
_no domatia_ = 0.26±0.26 SE; F_1, 25_ = 0.69, P = 0.41). Study type again accounted for substantial variation in the system: experimental studies had a smaller effect size than observational studies (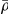
_obs_ - 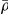
_exp_ = 0.35±0.17 SE; F_1, 25_ = 4.24, P = 0.05).

**Figure 3:**
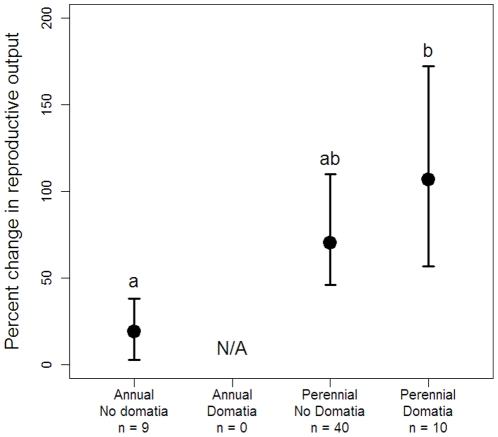
Benefits of ant presence varied according to plant life history and presence/absence of domatia. Predicted mean percent changes (±1 SE) in reproductive output when ants are present for the three combinations of domatia production and life history present in the dataset. Effects of domatia and life history combinations are population marginal means averaged over the effects of observational and experimental studies. Letters indicate significant pairwise differences (p<0.05) between groups based on Tukey's post-hoc multiple comparisons of means.

Importantly, no annual plant species in our analysis produced domatia, thus confounding the effects of plant life history and domatia. In fact, we do not know of any annual plant species that produce domatia. Therefore, to evaluate the effects of these two factors, we analyzed a mixed-effects model containing a composite variable with three levels representing the combinations of domatia production and plant life history present in our dataset. This variable explained a significant amount of variation among studies (F_2, 24_ = 3.63, P = 0.042). Specifically, the reproductive output of domatia-bearing, perennial plants benefited substantially more from ant protection than that of annual, non-domatia-bearing plants (Tukey's HSD, P = 0.026), but the effect for perennial plants that did not produce domatia was not significantly different from either of the other groups (Fig. 3). Experimental studies had a smaller effect size than observational studies across all three domatia-life history combinations (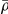
_obs_ - 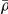
_exp_ = 0.33±0.15 SE; F_1, 25_ = 4.99, P = 0.035).

Nearly all of the ant-plants in our study produced extrafloral nectaries (26 of the 28 species) and only two produced food bodies, so we were unable to test the effects of these two nutritional rewards on the efficacy of ant defense. Of the plant species that produced EFN, 15 produced nectaries on the reproductive structures, most commonly on the floral bracts or calyx. There was no difference in the effects of ant protection on plant reproductive output between plants with EFN on the reproductive structures and those with EFN on vegetative parts (F_1, 25_ = 0.32, P = 0.58). Honeydew-producing trophobionts were recorded in 12 of the ant and plant species combinations, but their presence had no effect on the degree to which ant presence influenced plant reproductive output (F_1, 30_<0.001, P>0.99). Ant effects on plant reproduction did not vary significantly among plant growth forms (F_5, 21_ = 0.73, P = 0.61). Study type was significant in the analysis of EFN location (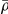
_obs_ - 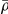
_exp_ = 0.42±0.17 SE; F_1, 25_ = 6.15, P = 0.02), but not in the tests for effects of trophobionts or plant growth form (F_1, 26_ = 3.67, P = 0.067; F_1, 21_ = 1.03, P = 0.32, respectively).

### Effects of ant characteristics on mutualism strength

The number of ant taxa associated with the plant species in the studies included in our analysis ranged from 1 to 34 (mean  = 6.61±1.22 SE). Plant species that produced domatia (6 of the 28 species) were associated with fewer ant partners than plants that did not produce domatia (domatia: 2.0 ant species ±2.80 SE, no domatia: 7.86 ant species ±1.30 SE; F_1, 26_ = 4.38, P = 0.046). There was no relationship between the effect of ants on reproductive output and the number of ant partners (analyzed using the reciprocal of number of ant species, F_1, 30_ = 0.23, P = 0.64). As domatia-bearing plants have fewer ant partner species, we also analyzed a model with an interaction term for domatia and the number of ant species to elucidate the potential conditional effects of these variables. However, there was no effect of domatia, ant species, or their interation (inverse of number of ant species: F_1, 30_ = 0.24, P = 0.63; domatia: F_1, 24_ = 1.07, P = 0.31; interaction: F_1, 24_ = 0.39, P = 0.54). Observational studies had marginally larger effect sizes than experimental studies in both the analysis of number of ant species (F_1, 26_ = 3.77, P = 0.063) and in the model that included number of ant species and domatia (F_1, 24_ = 4.09, P = 0.054).

We found no differences in protective effect among formicines, dolichoderines and myrmecines (F_2, 16_ = 1.34, P = 0.29), the three most common dominant subfamilies of ants in the dataset. The most common ant species (identified from 36 studies) ranged in body length from 1.3 mm (*Wasmannia auropunctata*) to 8.2 mm (*Ectatomma tuberculatum*), but there was no effect of ant size on the benefits of ant presence for plant reproduction (F_1, 19_ = 0.57, P = 0.46). To test Rico-Gray and Oliveira's [14] hypothesis that larger ants confer greater protective benefits for myrmecophilic plants that do not produce domatia, we also analyzed a model with both ant size and presence of domatia as predictor variables. In contrast to their prediction, the interaction between these factors was not significant (F_1, 17_ = 0.03, P = 0.87) and the main effects in this model corroborated our previous analyses (ant size: F_1, 17_ = 0.58, P = 0.46; domatia: F_1, 17_ = 3.78, P = 0.068). These results suggest that larger ant species did not have greater benefits for plant reproduction across the ant-plant systems included in our study. There was no effect of study type (experimental vs. observational) in these three analyses (0.24<P<0.51).

### Effects of environmental characteristics on mutualism strength

The effect of ants on plant reproduction was unaffected by annual precipitation (F_1, 30_ = 2.5, P = 0.12) or habitat type (F_2, 29_ = 0.42, P = 0.66). The effect size in observational studies was significantly greater than that for experimental studies in both analyses (F_1, 26_ = 9.80, P = 0.0043 and F_1, 26_ = 6.02, P = 0.02, respectively). A model testing the interaction term for these two environmental variables also found no significant effects of either variable or their interaction, suggesting that there was no systematic effect of abiotic variables across ant-plant systems (habitat: F_2, 26_ = 0. 026, P = 0.97; precipitation: F_1, 26_ = 2.16, P = 0.15; interaction: F_2, 26_ = 2.51, P = 0.10; study type: F_1, 26_ = 2.09, P = 0.14).

## Discussion

### Effects of ant protection on herbivory and plant reproduction

The evolution and maintenance of mutualisms requires that the relationship results in net fitness benefits for the interacting species. However, many empirical studies of ant-plant mutualisms examine foliar herbivory as a proxy for plant fitness [2]. Measuring fitness is challenging, but other surrogates (such as reproduction) are likely more closely related to fitness than more indirect measures such as herbivory or herbivore abundance [35]. Chamberlain and Holland [27] found no correlation between the effects of ant protection on herbivory and plant “performance” (consisting primarily of studies of reproduction with a small number of studies that measured plant growth). In contrast to their results, we found a significant positive correlation between these two responses, with the mean effect on herbivory greater than the effect on plant reproduction. Our finding is consistent with Schmitz et al.'s [12] conclusion that the cascading effect of predator removal for plant reproductive output is attenuated compared to the effects on herbivore abundance or herbivory. Unfortunately, there were not enough primary studies that reported responses of both reproductive and foliar damage to assess the effects of alternative plant defensive strategies, differences in ant behavior or other ecological variables on variation in the relationship between these two measures of ant defense.

Although the results of our analyses differed, our findings and those of Chamberlain and Holland [27] together suggest that herbivory responses may be poor proxies for effects on plant fitness – ant-plant mutualisms may be more (or less) beneficial for plant fitness than would be inferred by quantifying only herbivory. Several distinct mechanisms could produce this disparity. First, as has been found for the ant guards of *Acacia drepanolobium*, ants may protect vegetative structures but not reproductive structures from herbivore damage [46] (but see [25]). Second, mutualistic ants may protect reproductive structures from damage but also reduce plant reproductive output by disrupting pollination [47]. Understanding the effects of floral volatiles and nectar on ant protection of plant reproductive parts is an active area of research that may improve our understanding of the causes and consequences of variation in ant-plant protection mutualisms [3].

Additionally, plant tolerance to herbivory may explain the greater effects of ant defense on foliar damage than on reproductive output suggested by our analysis. Many plant species increase allocation of resources to reproduction in response to foliar damage [48]–[50]. Such a strategy could contribute to the evolution and maintenance of ant-plant mutualisms in at least two ways: 1) reducing the fitness costs of association with low-quality partners or occasional antagonistic interactions with otherwise high-quality partners, and 2) minimizing the effects of short-term partner absence [51], [52]. Notably, the “snapshot” understanding gained from most experimental studies may not fully capture the long-term effects of tolerance strategies for plants because there is a limit to the resources available for reproduction under long-term defoliation. Therefore, our results (given the design of available primary studies) may have underestimated lifetime fitness benefits of ant protection.

### Effects of plant, ant, environmental, and study traits on plant reproduction

We generally found a larger effect size for observational versus experimental studies. At least two explanations for this difference seem possible. First, the plants without ants in observational studies may have been without their biotic defenders for a long period (longer than the duration of the ant removal experiments), and therefore suffered greater long-term negative effects from herbivory. However, Chamberlain and Holland [27] found no effect of length of study on the effects of ant absence in their study (but see caveats raised by Osenberg et al. [29]). Second, presence or absence of ants in the observational studies could have been related to host plant health, with untended plants already having lower reproductive output for reasons unrelated to effects of mutualistic ants per se (e.g., [8]). This type of positive, causal relationship between habitat quality and colonization/survival is likely common in natural systems [53], [54], and suggests that observational and experimental approaches are both valuable but may produce very different results.

We found only one ecological factor, plant life history, which significantly explained variation across systems in the reproductive benefit plants gained from ant protection. Neither Chamberlain and Holland [27] nor Rosumek et al. [28] explicitly examined this variable, although its influence may be ultimately responsible for some of the differences among systems that they attributed to other plant traits that are likely correlated with annual or perennial life histories. For example, because only perennial plants produced domatia, it is difficult to isolate the separate contributions of these two variables. Indeed, it is likely that Chamberlain and Holland [27] and Rosumek et al. [28] overestimated the role of domatia because they were unable to decouple plant life history and domatia in their analyses. In contrast, we examined both traits separately and together, and found a demonstrable effect of life history but not domatia. Annual plant species generally allocate fewer resources to anti-herbivore defense such as secondary chemistry than perennial species [50], [55], and our results indicate that this pattern may extend to indirect biotic defenses such as protective ants. However, the small number of plant species that produced domatia, and the confounding of plant life history and domatia presence, limited our analysis and complicated interpretation of either variable.

The evolution of EFN on or near reproductive structures in some ant-plants suggests that they confer a fitness benefit, but previous research has been equivocal about their effects [25], [46], [47]. We found no difference in ant protection between plants with EFN on the vegetative versus reproductive structures. This could result from differing effects of EFN location among systems, or it could indicate that EFN serve other purposes, such as attracting floral visitors or other natural enemies of herbivores such as parasitoid or predatory wasps [56], [57]. We also investigated the effects of honeydew-producing insects on the protective efficacy of ants. Ant-associated Hemiptera and Lepidoptera feeding on plant tissue clearly represent a cost to the plant and benefit ants by producing sugar-rich honeydew [23], [24], [58], but our study did not indicate that this cost is associated with more effective ant protection of plants. Ants commonly tend cryptic Hemiptera, such as Coccidae and Pseudococcidae, within domatia [59], [60], so it is possible that the occurrence of honeydew-producing trophobionts in myrmecophytic systems was underreported, thus obscuring possible effects in our analyses. Further investigations into the effects of honeydew production and consumption in ant-plant protective systems are required to elucidate these widespread but poorly understood relationships.

Association with multiple ant species may be risky for ant-plants because diffuse interactions with low-quality partners may result in reduced net fitness benefits compared to more specialized relationships with high-quality mutualists [61], [62]. Contrary to our expectation that the benefit gained from ant protection for plant reproduction would decrease as the number of ant partners increased, we found that the number of ant partners did not influence the effects of ants on plant reproductive output. This result corroborates the findings of Chamberlain and Holland [27] and Rosumek et al. [28], although both studies did find the expected pattern in their analyses of herbivory effect size. Several studies have found that high-quality ant partners are also the most abundant (e.g., [17], [62]), so our result could be confounded by the relationship between relative abundance of ant species and their protective efficacy. Such unequal and non-random interaction frequencies between alternative ant partners and the host plant could produce a net positive effect of ant association, thus maintaining the mutualistic relationship [63]–[65].

Ant-plant protective mutualisms are most diverse and complex in tropical forests [2] where herbivory rates are higher than in temperate forests [66]. Because annual precipitation is associated with both latitude and vegetation type, we predicted that the effect of ant protection would increase with precipitation and could also vary among habitat types. However, we did not find a significant effect of precipitation, habitat or their interaction on the importance of ant protection for plant reproductive output. Chamberlain and Holland [27] found a similar result to ours when examining plant reproduction, although they did find significant effects when examining herbivory. In contrast, Rosumek et al. [28] found differences between tropical and temperate systems in measures of both herbivory and reproduction, possibly because they used a dichotomous categorization of latitude (tropical v. temperate) rather than finer-scale categorical or continuous characterization of habitat.

### Methodological considerations

The literature search protocol we used appears similar to that used by Chamberlain and Holland [27] and Rosumek et al. [28], but our criteria for selection of primary studies in our analysis differed. We only included papers that directly measured plant reproduction in response to presence or absence of presumably mutualistic ant guards. Therefore, we excluded primary studies that identified the ant partners as parasites *a priori*. We also excluded studies in which the only ant reward was produced by honeydew-producing Hemiptera rather than directly by the plant. Both of these types of relationships were included in the meta-analyses by Chamberlain and Holland [27] and Rosumek et al. [28]. It is unclear how differences in literature searches and study selection may have affected the three sets of results, but it is likely that ants that parasitize ant-plants are detrimental or not as beneficial to reproductive output of the host plant [36], [37]. Therefore, under similar analysis, we would expect our study to show a higher effect of ant presence on plant reproduction than found in the other two studies.

Many meta-analytic approaches remain relatively simplistic, often relying on univariate, single-factor, non-hierarchical statistical tests even when the systems are complex and include multiple causal factors that may be correlated (e.g., [34]). Previous meta-analyses of ant-plant mutualisms dealt with multiple studies on the same species in two different ways, both of which have limitations. Treating all studies as independent samples [12], [28] is problematic because there are many sources of non-independence among studies that may lead to bias (if some systems are over-represented in the data) or underestimation of uncertainty (even if sampling bias per se is lacking). Aggregating all data from a single species into a pooled effect size (e.g., [27]) is also problematic because it disregards the among-study, within-species variability, which is an important component of total variance in mixed effects meta-analysis [39]. Furthermore, neither of these techniques allows analysis of variation within plant species (e.g., due to differences in habitat among study sites or ant partners within study sites). Our analytic approach addressed these problems by accounting for non-independence of samples from the same plant species and incorporating the among-study, within-species, variance. Consequently, we were able to analyze the effects of traits of different ant species that interacted with the same plant species, although in our study this did not produce any additional insights into factors that account for variation in plant protection.

Traditional experimental studies often use factorial designs to evaluate the main and interactive effects of multiple predictor variables. Such approaches are rare in ecological meta-analyses for at least two reasons: 1) standard software (e.g., MetaWin [67]) cannot analyze factorial designs with multiple predictor variables; 2) meta-analyses are opportunistic and not design-based – because few of the summarized experiments are themselves crossed, the factorial nature of the analyses arises from sampling rather than planning. Our methodology allowed us to examine multiple predictor variables in the same statistical model, but this analytic flexibility was limited by available data. For example, we analyzed the interaction between some predictor variables (e.g., domatia and ant size) with factorial designs when the data permitted, but sample sizes were quite low and variation among systems was high. We also addressed confounding of variables that were not fully crossed (e.g., plant life history and domatia) by analyzing the effects of each variable separately and by testing for correlation among predictor variables and then incorporating this knowledge into our model-building procedure and our interpretation of results. In our study, this led to recognized uncertainty in interpretation of the effects of plant life history and domatia production on ant-plant mutualisms and suggested that recent studies examining these systems may have misidentified the causes of variation in effect sizes. Furthermore, our work clarifies the need for future studies to directly investigate the role of these plant traits on ant protection in ant-plant mutualisms.

## Supporting Information

Table S1 Primary studies included in meta-analysis and effect sizes.
(0.02 MB DOCX)

Appendix S1 Methods used for literature search and data extraction.
(0.11 MB DOC)

Appendix S2 R code for variance-weighted correlation analysis.
(0.01 MB DOCX)
