# Supplementary material for: Benefits for Plants in Ant-Plant Protective Mutualisms: A Meta-Analysis
Source: PLoS One. 2010 Dec 22;5(12):e14308. doi: 10.1371/journal.pone.0014308 (PMC3008678; doi:10.1371/journal.pone.0014308)
Supplement: Table S1 — Primary studies included in meta-analysis and effect sizes. (0.02 MB DOCX) [file pone.0014308.s001.docx]

**Benefits for plants in ant-plant protective mutualisms: A meta-analysis**

**Table S2. Primary studies included in meta-analysis and effect sizes.**

Plant species, ant species and effect sizes for ant presence on reproductive output and herbivore damage from the 59 primary studies used in our meta-analysis. The most abundant ant species is listed for studies in which they could be identified, but in other studies a mixed assemblage of ants tended the plants for which the authors did not designate a numerically dominant species.

|  |  |  | **Effect size ± σ^2^** | |  |
| --- | --- | --- | --- | --- | --- |
| **Paper** | **Plant species** | **Ant species** | **Herbivory** | **Reproduction** | **Citation** |
| Barton 1986 | *Cassia* (= *Chamaecrista*) *fasciculata* | *Iridomyrmex pruinosum*  (= *Forelius pruinosus*) and others | 0.044 ± 0.061 | 0.50 ± 0.12 | Ecology, 67, 495-504 |
| " | *Cassia* (= *Chamaecrista*) *fasciculata* | *Crematogaster clara* and others | -0.059 ± 0.28 | 0.30 ± 0.046 | " |
| Bentley 1977 | *Bixa orellana* | Mixed assemblage |  | 1.44 ± 2.23 | J.Ecol., 65, 27-38 |
| Boeklen 1984 | *Cassia* (= *Chamaecrista*) *fasciculata* | Mixed assemblage |  | 0.22 ± 0.040 | Ecol.Entomol., 9, 243-249 |
| Del Claro et al. 1996 | *Qualea multiflora* | Mixed assemblage | 0.62 ± 0.031 | 0.58 ± 0.039 | J.Trop.Ecol., 12, 887-892 |
| Devall & Thien 1989 | *Ipomoea pes-caprae* | Not stated |  | 1.19 ± 0.44 | Am.J.Bot., 76, 1821-1831 |
| " | *Ipomoea pes-caprae* | Not stated |  | 0.58 ± 0.26 | " |
| " | *Ipomoea pes-caprae* | Not stated |  | 1.05 ± 0.23 | " |
| " | *Ipomoea pes-caprae* | Not stated |  | 0.34 ± 0.16 | " |
| " | *Ipomoea pes-caprae* | Not stated |  | 0.29 ± 0.068 | " |
| Fisher 1992 | *Caularthron bilamellatum* | *Azteca velox* |  | 0.32 ± 0.10 | J.Trop.Ecol., 8, 109-114 |
| Freitas et al. 2000 | *Croton sacropetalus* | *Zacrypocerus* sp. and others | 1.98 ± 0.57 | 0.21 ± 0.091 | Flora, 195, 398-402 |
| Gaume et al. 2005 | *Humboltia brunonis* | *Technomyrmex albipes* | 1.75 ± 1.26 | 1.87 ± 1.18 | Evol.Ecol.Res., 7, 435-452 |
| Horvitz & Schemske 1984 | *Calathea ovandensis* | Mixed assemblage |  | 0.37 ± 0.014 | Ecology, 65, 1369-1378 |
| " | *Calathea ovandensis* | *Wasmannia auropunctata* and others |  | 0.93 ± 0.017 | " |
| " | *Calathea ovandensis* | *Crematogaster sumichrasti* and others |  | 0.56 ± 0.014 | " |
| " | *Calathea ovandensis* | *Solenopsis geminata* and others |  | 0.37 ± 0.028 | " |
| " | *Calathea ovandensis* | *Brachymyrmex musculus* and others |  | 0.36 ± 0.0010 | " |
| " | *Calathea ovandensis* | *Monacis bispinosus* and others |  | 0.34 ± 0.068 | " |
| " | *Calathea ovandensis* | *Paratrechina* sp. and others |  | 0.32 ± 0.036 | " |
| " | *Calathea ovandensis* | *Pachycondyla unidentata* and others |  | 0.095 ± 0.046 | " |
| " | *Calathea ovandensis* | *Pheidole gouldi* and others |  | -0.076 ± 0.058 | " |
| Kelly 1986 | *Cassia* (= *Chamaecrista*) *fasciculata* | Mixed assemblage | 0.13 ± 0.022 | -0.28 ± 0.021 | Oecologia, 69, 600-605 |
| " | *Cassia* (= *Chamaecrista*) *fasciculata* | Mixed assemblage | 0.19 ± 0.52 | 0.42 ± 0.040 |  |
| Koptur 1998 | *Vicia angustifolia* | *Iridomyrmex humilis*  (= *Linepithema humile*) | 1.52 ± 0.027 | 0.38 ± 0.021 | Am.J.Bot., 66, 1016-1020 |
| Letourneau 1998 | *Piper sagittifolium* | *Pheidole bicornis* |  | 0.62 ± 0.055 | Ecology, 79, 593-603 |
| McLain 1983 | *Passiflora incarnata* | Mixed assemblage | 1.64 ± 0.13 | 1.28 ± 0.047 | Am.Midl.Nat., 110, 433-439 |
| Miller 2007 | *Opuntia imbricata* | *Limeotopum apiculatum* | 0.81 ± 0.11 | 0.28 ± 0.012 | Oikos, 116, 500-512 |
| " | *Opuntia imbricata* | *Crematogaster opuntiae* | 0.087 ± 0.16 | 0.11 ± 0.023 | " |
| Ness et al. 2006 | *Ferocactus wislizeni* | *Crematogaster opuntiae* |  | 0.51 ± 0.091 | Ecology, 87, 912-921 |
| " | *Ferocactus wislizeni* | *Forelius* sp. |  | 0.56 ± 0.19 | " |
| " | *Ferocactus wislizeni* | *Solenopsis aurea* |  | 0.57 ± 0.18 | " |
| " | *Ferocactus wislizeni* | *Solenopsis xyloni* |  | 0.62 ± 0.18 | " |
| " | *Ferocactus wislizeni* | Mixed assemblage |  | 0.56 ± 0.18 | " |
| O’Dowd and Catchpole 1983 | *Helichrysum viscosum* | *Iridomyrmex purpureus* and others |  | 0.045 ± 0.0088 | Oecologia, 59, 191-200 |
| " | *Helichrysum bracteatum* | *Iridomyrmex* spp. |  | 0.039 ± 0.0097 | " |
| Oliveira 1997 | *Caryocar brasiliense* | *Camponotus* spp. and others | 1.15 ± 0.17 | 0.16 ± 0.28 | Funct.Ecol., 11, 323-330 |
| Oliveira et al. 1999 | *Opuntia stricta* | *Camponotus planatus* and others | 2.13 ± 0.75 | 0.41 ± 0.014 | Funct.Ecol., 13, 623-631 |
| Rico-Gray & Thien 1989a | *Schomburgkia tibicinis* | *Crematogaster brevispinosa* |  | -1.35 ± 2.0 | Oecologia, 81, 487-489 |
| Rico-Gray & Thien 1989b | *Schomburgkia tibicinis* | *Camponotus planatus* |  | -0.12 ± 2.0 | J.Trop.Ecol., 5, 109-112 |
| " | *Schomburgkia tibicinis* | *Camponotus abdominalis* |  | 0.85 ± 2.0 | " |
| " | *Schomburgkia tibicinis* | *Camponotus rectangularis* |  | 1.26 ± 2.0 | " |
| " | *Schomburgkia tibicinis* | *Ectatomma tuberculatum* |  | 1.30 ± 2.0 | " |
| Rudgers 2004 | *Gossypium thurberi* | *Forelius pruinosus* and others | 1.14 ± 0.14 | 0.41 ± 0.076 | Ecology, 85, 192-205 |
| Ruhren 2003 | *Chamaecrista nictitans* | Mixed assemblage | -0.29 ± 0.36 | -0.041 ± 0.0089 | Plant Ecol., 166, 189-198 |
| Rutter & Rausher 2004 | *Chamaecrista fasciculata* | Mixed assemblage | 0.036 ± 0.48 | -0.36 ± 0.10 | Evolution, 58, 2657-2668 |
| Schemske 1980 | *Costus woodsonii* | *Camponotus planatus* and others |  | 1.06 ± 0.10 | J.Ecol., 68, 959-967 |
| " | *Costus woodsonii* | *Wasmannia auropunctata* and others |  | 1.21 ± 0.015 | " |
| Sobrinho et al. 2002 | *Triumfetta semitriloba* | Mixed assemblage |  | 0.48 ± 0.037 | Sociobiology, 39, 353-368 |
| Stephenson 1982 | *Catalpa speciosa* | Mixed assemblage |  | 0.27 ± 0.020 | Ecology, 63, 663-669 |
| Torres-Hernandez et al. 2000 | *Turnera ulmifolia* | Mixed assemblage | 0.48 ± 0.022 | 1.24 ± 0.050 | Acta Zool.Mex., 81, 13-21 |
| " | *Turnera ulmifolia* | *Camponotus planatus* |  | -0.38 ± 0.12 | " |
| " | *Turnera ulmifolia* | Mixed assemblage |  | -0.68 ± 0.17 | " |
| " | *Turnera ulmifolia* | *Conomyrma* (= *Dorymyrmex*) sp. |  | -0.89 ± 0.11 | " |
| " | *Turnera ulmifolia* | *Camponotus abdominalis* |  | 0.56 ± 0.046 | " |
| Vasconcelos 1991 | *Maieta guianensis* | *Pheidole minutula* | 3.56 ± 0.078 | 3.81 ± 13 | Oecologia, 95, 439-443 |
| Vesprini et al. 2003 | *Dyckia floribunda* | Mixed assemblage |  | 0.62 ± 0.052 | Can.J.Bot., 81, 24-27 |
| Wagner 1997 | *Acacia constricta* | *Formica perpilosa* | 0.17 ± 0.18 | 0.63 ± 0.16 | J.Ecol., 85, 83-93 |
| Willmer & Stone 1997 | *Acacia zanzibarica* | *Crematogaster* spp. |  | 0.87 ± 0.58 | Nature, 388, 165-167 |
