# Supplementary material for: Benefits for Plants in Ant-Plant Protective Mutualisms: A Meta-Analysis
Source: PLoS One. 2010 Dec 22;5(12):e14308. doi: 10.1371/journal.pone.0014308 (PMC3008678; doi:10.1371/journal.pone.0014308)
Supplement: Appendix S1 — Methods used for literature search and data extraction. (0.11 MB DOC) [file pone.0014308.s002.doc]

**Electronic supplementary material**

**Trager et al. 2010. Benefits for plants in ant-plant protective mutualisms: A meta-analysis**

**Appendix I. Methods used for literature search and data extraction**

*Literature search.* We conducted identical Boolean searches to locate publications that included the keywords “ant-plant” or “myrmecophyte” and also contained “repro*”, “fecundity”, “seed”, “fruit” or “flower” with several common electronic databases (Zoological Record, Wilson Web, Web Siris/CAB Abstracts, Web of Science, Ebscohost/Academic Search Premier, Wilson Web/Bio+Agricultural Index Plus, CSA Illumina Biological Sciences, ISI Biosis Previews). We identified several more publications from the references cited in these studies.

*Data extraction and derivation of means and variances.* We used freely available digitization and data extraction software to extract data from figures when they were not reported in text or tables (GetData Graph Digitizer v. 2.22, copyright S. Fedorov, 2002-2006; ImageJ v. 1.37, copyright Wayne Rasband, National Institutes of Health, USA). A small number of studies did not present results for which we could extract means and variances (e.g., they reported medians and quartiles or means and ranges instead); for these we calculated conservative estimates for these parameters using Chebyshev’s inequality (i.e., for most distributions no more than 1/*k*^2^ of the samples are farther than *k* standard deviations from the sample mean [1]). One paper [2] presented ant abundance as a continuous variable; for the ant absence treatment we calculated the regression estimates of the *y*-intercept and its variance and for the ant presence treatment we calculated plant reproductive output and its variance at mean ant abundance.

*Data on ant and plant traits.* For papers that did not provide information on all of these variables, we consulted additional sources that described the natural history of the focal plant species and their interactions with ants. All data on ant size was obtained by estimating the total body length of worker ants from freely-available, high-resolution images on AntWeb or J.T. Longino’s Ants of Costa Rica site [3]. We obtained precipitation data for the study sites from archived data available online from the National Climatic Data Center (National Oceanic and Atmospheric Administration, United Stated Department of Commerce).

**References**

1. Grimmett GR, Stirzaker DR (2001) Probability and random processes. USA: Oxford University Press.

2. Willmer PG, Stone GN (1997) How aggressive ant-guards assist seed-set in *Acacia* flowers. Nature 388: 165-167.

3. Longino JT (2009) Ants of Costa Rica. Available: <http://academic.evergreen.edu/projects/ants/AntsOfCostaRica.html> via the Internet.

**Appendix II. R code for variance-weighted correlation analysis.**

As described in the Methods section, we tested the relationship between effect sizes for plant reproduction and herbivory with a weighted correlation analysis using studies that provided data on both responses. The values for these variables can be found in Appendix III. The annotated R code used in this analysis is below, with code in blue:

1. Perform simple linear regression to obtain starting values for maximum likelihood function defined below:

fit1 <- lm(Repro.Effect ~ Herb.Effect, data = dat)

where Repro.Effect is the effect size of ants on plant reproductive output and Herb.Effect is the effect size of ants on foliar herbivory.

2. Specify model and likelihood estimation of parameters:

likfun <- function(a, b) {

yhat <- a + b * x

xhat <- (y - a)/b

sum((x - xhat)^2/v.x + (y - yhat)^2/v.y)/2

}

inits <- as.list(coef(fit1))

names(inits) <- c("a", "b")

where a and b indicate the intercept and slope terms, x and y indicate the reproduction and herbivory effect sizes and v.x and v.y indicate the variance for each effect size.

3. Load appropriate R package for maximum likelihood estimation procedure and define the variable set from data file:

library(bbmle)

dat2 <- with(dat, data.frame(x = Herb.Effect, y = Repro.Effect, v.x = Herb.Var, v.y = Repro.Var))

4. The estimate of the intercept term (a) in the first iteration of model fitting with function mle2 were very close to 0, so we removed that parameter from the model to obtain a more precise fit for the primary question of the relationship between the reproduction and herbivory effect sizes. After removing the intercept term, the model fitting was specified as follows:

fit <- mle2(likfun, start = list(a = 0, b = 1), fixed = list(a = 0), data = dat2)

5. Call for estimate of slope term and confidence intervals:

coef(fit)

confint(fit, quiet = TRUE)

**Appendix III. Primary studies included in meta-analysis and effect sizes.**

Plant species, ant species and effect sizes for ant presence on reproductive output and herbivore damage from the 59 primary studies used in our meta-analysis. We name the most abundant ant species when available, but in other studies a mixed assemblage of ants tended the plants for which the authors did not designate a numerically dominant species.

|  |  |  | **Effect size ± σ^2^** | |  |
| --- | --- | --- | --- | --- | --- |
| **Paper** | **Plant species** | **Ant species** | **Herbivory** | **Reproduction** | **Citation** |
| Barton 1986 | *Cassia* (= *Chamaecrista*) *fasciculata* | *Iridomyrmex pruinosum*  (= *Forelius pruinosus*) and others | 0.044 ± 0.061 | 0.50 ± 0.12 | Ecology, 67, 495-504 |
| " | *Cassia* (= *Chamaecrista*) *fasciculata* | *Crematogaster clara* and others | -0.059 ± 0.28 | 0.30 ± 0.046 | " |
| Bentley 1977 | *Bixa orellana* | Mixed assemblage |  | 1.44 ± 2.23 | J.Ecol., 65, 27-38 |
| Boeklen 1984 | *Cassia* (= *Chamaecrista*) *fasciculata* | Mixed assemblage |  | 0.22 ± 0.040 | Ecol.Entomol., 9, 243-249 |
| Del Claro et al. 1996 | *Qualea multiflora* | Mixed assemblage | 0.62 ± 0.031 | 0.58 ± 0.039 | J.Trop.Ecol., 12, 887-892 |
| Devall & Thien 1989 | *Ipomoea pes-caprae* | Not stated |  | 1.19 ± 0.44 | Am.J.Bot., 76, 1821-1831 |
| " | *Ipomoea pes-caprae* | Not stated |  | 0.58 ± 0.26 | " |
| " | *Ipomoea pes-caprae* | Not stated |  | 1.05 ± 0.23 | " |
| " | *Ipomoea pes-caprae* | Not stated |  | 0.34 ± 0.16 | " |
| " | *Ipomoea pes-caprae* | Not stated |  | 0.29 ± 0.068 | " |
| Fisher 1992 | *Caularthron bilamellatum* | *Azteca velox* |  | 0.32 ± 0.10 | J.Trop.Ecol., 8, 109-114 |
| Freitas et al. 2000 | *Croton sacropetalus* | *Zacrypocerus* sp. and others | 1.98 ± 0.57 | 0.21 ± 0.091 | Flora, 195, 398-402 |
| Gaume et al. 2005 | *Humboltia brunonis* | *Technomyrmex albipes* | 1.75 ± 1.26 | 1.87 ± 1.18 | Evol.Ecol.Res., 7, 435-452 |
| Horvitz & Schemske 1984 | *Calathea ovandensis* | Mixed assemblage |  | 0.37 ± 0.014 | Ecology, 65, 1369-1378 |
| " | *Calathea ovandensis* | *Wasmannia auropunctata* and others |  | 0.93 ± 0.017 | " |
| " | *Calathea ovandensis* | *Crematogaster sumichrasti* and others |  | 0.56 ± 0.014 | " |
| " | *Calathea ovandensis* | *Solenopsis geminata* and others |  | 0.37 ± 0.028 | " |
| " | *Calathea ovandensis* | *Brachymyrmex musculus* and others |  | 0.36 ± 0.0010 | " |
| " | *Calathea ovandensis* | *Monacis bispinosus* and others |  | 0.34 ± 0.068 | " |
| " | *Calathea ovandensis* | *Paratrechina* sp. and others |  | 0.32 ± 0.036 | " |
| " | *Calathea ovandensis* | *Pachycondyla unidentata* and others |  | 0.095 ± 0.046 | " |
| " | *Calathea ovandensis* | *Pheidole gouldi* and others |  | -0.076 ± 0.058 | " |
| Kelly 1986 | *Cassia* (= *Chamaecrista*) *fasciculata* | Mixed assemblage | 0.13 ± 0.022 | -0.28 ± 0.021 | Oecologia, 69, 600-605 |
| " | *Cassia* (= *Chamaecrista*) *fasciculata* | Mixed assemblage | 0.19 ± 0.52 | 0.42 ± 0.040 |  |
| Koptur 1998 | *Vicia angustifolia* | *Iridomyrmex humilis*  (= *Linepithema humile*) | 1.52 ± 0.027 | 0.38 ± 0.021 | Am.J.Bot., 66, 1016-1020 |
| Letourneau 1998 | *Piper sagittifolium* | *Pheidole bicornis* |  | 0.62 ± 0.055 | Ecology, 79, 593-603 |
| McLain 1983 | *Passiflora incarnata* | Mixed assemblage | 1.64 ± 0.13 | 1.28 ± 0.047 | Am.Midl.Nat., 110, 433-439 |
| Miller 2007 | *Opuntia imbricata* | *Limeotopum apiculatum* | 0.81 ± 0.11 | 0.28 ± 0.012 | Oikos, 116, 500-512 |
| " | *Opuntia imbricata* | *Crematogaster opuntiae* | 0.087 ± 0.16 | 0.11 ± 0.023 | " |
| Ness et al. 2006 | *Ferocactus wislizeni* | *Crematogaster opuntiae* |  | 0.51 ± 0.091 | Ecology, 87, 912-921 |
| " | *Ferocactus wislizeni* | *Forelius* sp. |  | 0.56 ± 0.19 | " |
| " | *Ferocactus wislizeni* | *Solenopsis aurea* |  | 0.57 ± 0.18 | " |
| " | *Ferocactus wislizeni* | *Solenopsis xyloni* |  | 0.62 ± 0.18 | " |
| " | *Ferocactus wislizeni* | Mixed assemblage |  | 0.56 ± 0.18 | " |
| O’Dowd and Catchpole 1983 | *Helichrysum viscosum* | *Iridomyrmex purpureus* and others |  | 0.045 ± 0.0088 | Oecologia, 59, 191-200 |
| " | *Helichrysum bracteatum* | *Iridomyrmex* spp. |  | 0.039 ± 0.0097 | " |
| Oliveira 1997 | *Caryocar brasiliense* | *Camponotus* spp. and others | 1.15 ± 0.17 | 0.16 ± 0.28 | Funct.Ecol., 11, 323-330 |
| Oliveira et al. 1999 | *Opuntia stricta* | *Camponotus planatus* and others | 2.13 ± 0.75 | 0.41 ± 0.014 | Funct.Ecol., 13, 623-631 |
| Rico-Gray & Thien 1989a | *Schomburgkia tibicinis* | *Crematogaster brevispinosa* |  | -1.35 ± 2.0 | Oecologia, 81, 487-489 |
| Rico-Gray & Thien 1989b | *Schomburgkia tibicinis* | *Camponotus planatus* |  | -0.12 ± 2.0 | J.Trop.Ecol., 5, 109-112 |
| " | *Schomburgkia tibicinis* | *Camponotus abdominalis* |  | 0.85 ± 2.0 | " |
| " | *Schomburgkia tibicinis* | *Camponotus rectangularis* |  | 1.26 ± 2.0 | " |
| " | *Schomburgkia tibicinis* | *Ectatomma tuberculatum* |  | 1.30 ± 2.0 | " |
| Rudgers 2004 | *Gossypium thurberi* | *Forelius pruinosus* and others | 1.14 ± 0.14 | 0.41 ± 0.076 | Ecology, 85, 192-205 |
| Ruhren 2003 | *Chamaecrista nictitans* | Mixed assemblage | -0.29 ± 0.36 | -0.041 ± 0.0089 | Plant Ecol., 166, 189-198 |
| Rutter & Rausher 2004 | *Chamaecrista fasciculata* | Mixed assemblage | 0.036 ± 0.48 | -0.36 ± 0.10 | Evolution, 58, 2657-2668 |
| Schemske 1980 | *Costus woodsonii* | *Camponotus planatus* and others |  | 1.06 ± 0.10 | J.Ecol., 68, 959-967 |
| " | *Costus woodsonii* | *Wasmannia auropunctata* and others |  | 1.21 ± 0.015 | " |
| Sobrinho et al. 2002 | *Triumfetta semitriloba* | Mixed assemblage |  | 0.48 ± 0.037 | Sociobiology, 39, 353-368 |
| Stephenson 1982 | *Catalpa speciosa* | Mixed assemblage |  | 0.27 ± 0.020 | Ecology, 63, 663-669 |
| Torres-Hernandez et al. 2000 | *Turnera ulmifolia* | Mixed assemblage | 0.48 ± 0.022 | 1.24 ± 0.050 | Acta Zool.Mex., 81, 13-21 |
| " | *Turnera ulmifolia* | *Camponotus planatus* |  | -0.38 ± 0.12 | " |
| " | *Turnera ulmifolia* | Mixed assemblage |  | -0.68 ± 0.17 | " |
| " | *Turnera ulmifolia* | *Conomyrma* (= *Dorymyrmex*) sp. |  | -0.89 ± 0.11 | " |
| " | *Turnera ulmifolia* | *Camponotus abdominalis* |  | 0.56 ± 0.046 | " |
| Vasconcelos 1991 | *Maieta guianensis* | *Pheidole minutula* | 3.56 ± 0.078 | 3.81 ± 13 | Oecologia, 95, 439-443 |
| Vesprini et al. 2003 | *Dyckia floribunda* | Mixed assemblage |  | 0.62 ± 0.052 | Can.J.Bot., 81, 24-27 |
| Wagner 1997 | *Acacia constricta* | *Formica perpilosa* | 0.17 ± 0.18 | 0.63 ± 0.16 | J.Ecol., 85, 83-93 |
| Willmer & Stone 1997 | *Acacia zanzibarica* | *Crematogaster* spp. |  | 0.87 ± 0.58 | Nature, 388, 165-167 |
