# Supplementary material for: Benefits for Plants in Ant-Plant Protective Mutualisms: A Meta-Analysis
Source: PLoS One. 2010 Dec 22;5(12):e14308. doi: 10.1371/journal.pone.0014308 (PMC3008678; doi:10.1371/journal.pone.0014308)
Supplement: Appendix S2 — R code for variance-weighted correlation analysis. (0.01 MB DOCX) [file pone.0014308.s003.docx]

**Benefits for plants in ant-plant protective mutualisms: A meta-analysis**

**Appendix S2. R code for variance-weighted correlation analysis.**

As described in the Methods section, we tested the relationship between effect sizes for plant reproduction and herbivory with a weighted correlation analysis using studies that provided data on both responses. The values for these variables can be found in Appendix III. The annotated R code used in this analysis is below, with code in blue:

1. Perform simple linear regression to obtain starting values for maximum likelihood function defined below:

fit1 <- lm(Repro.Effect ~ Herb.Effect, data = dat)

where Repro.Effect is the effect size of ants on plant reproductive output and Herb.Effect is the effect size of ants on foliar herbivory.

2. Specify model and likelihood estimation of parameters:

likfun <- function(a, b) {

yhat <- a + b * x

xhat <- (y - a)/b

sum((x - xhat)^2/v.x + (y - yhat)^2/v.y)/2

}

inits <- as.list(coef(fit1))

names(inits) <- c("a", "b")

where a and b indicate the intercept and slope terms, x and y indicate the reproduction and herbivory effect sizes and v.x and v.y indicate the variance for each effect size.

3. Load appropriate R package for maximum likelihood estimation procedure and define the variable set from data file:

library(bbmle)

dat2 <- with(dat, data.frame(x = Herb.Effect, y = Repro.Effect, v.x = Herb.Var, v.y = Repro.Var))

4. The estimate of the intercept term (a) in the first iteration of model fitting with function mle2 were very close to 0, so we removed that parameter from the model to obtain a more precise fit for the primary question of the relationship between the reproduction and herbivory effect sizes. After removing the intercept term, the model fitting was specified as follows:

fit <- mle2(likfun, start = list(a = 0, b = 1), fixed = list(a = 0), data = dat2)

5. Call for estimate of slope term and confidence intervals:

coef(fit)

confint(fit, quiet = TRUE)
